# Diagnostic efficacy of fasting insulin-to-C-peptide ratio in exogenous insulin autoimmune syndrome

**DOI:** 10.3389/fmed.2026.1710461

**Published:** 2026-01-29

**Authors:** Tuo Han, Ziyue Wang, Jing Zhou, Nenghan Zhang, Jiajun Li, Na Feng, Chen Guo, Wenqian Zhang, Yongqin Li, Juanjuan Wu, Chunyan Zhang, Yan Zhang

**Affiliations:** 1 Department of Cardiovascular Medicine, The Second Affiliated Hospital of Xi'an Jiaotong University, Xi'an, China; 2 Institute of Cardiovascular Science, Translational Medicine Institute, Health Science Center, Xi'an Jiaotong University, Xi'an, China; 3 Department of Endocrinology, The Second Affiliated Hospital of Xi'an Jiaotong University, Xi'an, China

**Keywords:** diabetes mellitus, fasting C-peptide, fasting insulin, insulin autoimmune syndrome, receiver operating characteristic curve

## Abstract

**Background:**

The early-stage diagnosis of exogenous insulin autoimmune syndrome (EIAS) remains a significant clinical challenge for endocrinologists. This study aimed to investigate the diagnostic predictive utility of the fasting insulin-to-C-peptide ratio in EIAS.

**Methods:**

Diabetic patients with insulin autoantibody (IAA) testing admitted to our hospital between June 2023 and March 2024 were retrospectively enrolled. Participants were stratified into control and EIAS groups based on IAA status. A comparative analysis of EIAS clinical features was performed, with receiver operating characteristic (ROC) curve analysis used to determine the predictive efficacy of the fasting insulin/C-peptide ratio for EIAS.

**Results:**

Among 120 enrolled diabetic patients with IAA testing, 37 (30.8%) fulfilled the EIAS diagnostic criteria. Compared with patients in the control group, patients in the EIAS group were significantly older and had a longer duration of diabetes, demonstrated greater use of aspart insulin and recombinant human insulin formulations, and required higher insulin dosages. The EIAS cohort exhibited lower fasting glucose and HbA1c levels, alongside markedly elevated fasting and 2-h postprandial insulin concentrations and increased HOMA-IR values. ROC analysis established the fasting insulin/C-peptide ratio as a moderately accurate predictor of EIAS (AUC 0.802; 95% CI 0.724–0.880), with an optimal diagnostic threshold of 5.215 μU/ng providing 78.4% sensitivity and 77.1% specificity.

**Conclusion:**

Advanced age, prolonged diabetes duration, elevated insulin dosage requirements, recurrent hypoglycemia, and hyperinsulinemia constitute characteristic clinical features of EIAS. The fasting insulin/C-peptide ratio demonstrated favorable diagnostic performance, positioning it as a practical screening biomarker for EIAS.

## Introduction

Insulin autoimmune syndrome (IAS), also known as Hirata’s disease, represents a rare cause of hypoglycemia characterized by the production of autoantibodies targeting endogenous insulin, consequently inducing recurrent hypoglycemic episodes (1). Classic IAS is characterized by hyperinsulinemic hypoglycemia and elevated insulin autoantibody (IAA) titers, with no prior exposure to exogenous insulin (1, 2). The pathogenesis involves complex interactions between genetic susceptibility (e.g., HLA-DRB1*04:06 allele) and exogenous triggers (notably sulfhydryl-containing medication exposure), culminating in aberrant IAA binding and subsequent insulin release that disrupts glucose homeostasis (3, 4).

Over the past decade, increasing case reports described diabetic patients developing exogenous insulin-induced IAA with recurrent hypoglycemia mimicking classic IAS, a clinical entity termed exogenous insulin autoimmune syndrome (EIAS) (4, 5). The current evidence indicates comparable demographic profiles, insulin/C-peptide dynamics, spontaneous remission rates, therapeutic approaches, and prognoses between IAS and EIAS (6). However, the diagnosis of EIAS remains challenging, as it frequently mimics insulinoma or other hypoglycemic disorders clinically, underscoring the critical importance of enhanced disease recognition (7). Recent advances in immunoassay techniques have improved diagnostic detection rates, yet substantial knowledge gaps persist regarding its pathophysiology, therapeutic strategies, and long-term prognoses. In this study, we consecutively enrolled diabetic patients with IAA testing at our hospital to investigate the diagnostic predictive utility of the fasting insulin/C-peptide ratio in EIAS, thereby establishing a readily accessible and cost-effective predictive biomarker.

## Methods

### Study population

This study retrospectively enrolled diabetic patients with IAA testing results admitted to the Department of Endocrinology at our hospital between June 2023 and March 2024. The inclusion criteria comprised the following: (1) meeting diagnostic criteria for type 1 or type 2 diabetes mellitus (T1DM or T2DM) (8), including latent autoimmune diabetes in adults (LADAs) (9) and (2) availability of IAA testing results. Patients with insulinoma or duplicate cases were excluded. Ultimately, 120 diabetes patients were enrolled, with a mean age of 51.3 ± 17.5 years. The cohort included 68 male patients (56.7%), 97 T2DM patients (80.8%), 11 T1DM patients (9.2%), and 12 LADA patients (10.0%). The study protocol received approval from the Clinical Ethics Committee of the Second Affiliated Hospital of Xi’an Jiaotong University. All procedures adhered to the principles outlined in the Declaration of Helsinki. Written or oral informed consent was obtained from all participants.

### Data collection and covariates

Demographic characteristics (such as gender, age, and BMI), diabetes type, disease duration, insulin regimens and dosages, previous oral hypoglycemic agents, and laboratory parameters, including oral glucose tolerance test (OGTT) results, were collected. The homeostasis model assessment of insulin resistance (HOMA-IR) was calculated as follows: HOMA-IR = fasting glucose (mmol/L) × fasting insulin (μU/mL) / 22.5. Higher HOMA-IR values indicate greater severity of systemic insulin resistance.

### Diagnosis of EIAS

IAA testing was uniformly performed at Xi’an KingMed Diagnostic Laboratory. EIAS diagnosis was established based on discharge diagnoses retrieved from medical records by board-certified endocrinologists, incorporating clinical manifestations, laboratory findings, and positive IAA results, with the exclusion of alternative hypoglycemia etiologies (e.g., insulinoma and excessive hypoglycemic agents) (10). Diagnostic criteria comprised the following: (1) documented spontaneous hypoglycemia episodes with hyperinsulinemia and unsuppressed serum C-peptide; (2) positive IAA defined as either an insulin antibody binding rate of ≥5% by radioimmunoassay or IAA concentration of ≥20.00 RU/ml via chemiluminescence immunoassay; (3) received exogenous insulin injection; (4) radiographic exclusion of insulinoma or pancreatic pathology; and (5) no antecedent sulfhydryl-containing medication exposure or autoimmune comorbidity constituting supportive diagnostic evidence.

### Statistical analysis

Continuous variables were assessed for normality using the Shapiro–Wilk test. Normally distributed data are presented as mean ± standard deviation (SD) and are compared using independent samples *t*-tests. Non-normally distributed continuous variables are expressed as median with interquartile range (IQR) and analyzed using the Mann–Whitney U-test. Categorical variables are presented as frequencies (percentages) and compared using χ^2^ tests or Fisher’s exact test, as appropriate. Receiver operating characteristic (ROC) analysis was used to determine the efficacy of the fasting insulin to C-peptide ratio in predicting EIAS. The optimal diagnostic cutoff for the fasting insulin/C-peptide ratio was determined using the Youden index (J = Sensitivity + Specificity −1), which maximizes the discriminative ability of the biomarker.

All analyses were performed using R Statistical Software (Version 4.2.2, http://www.R-project.org, The R Foundation) and Free Statistics analysis platform (Version 1.9, Beijing, China, http://www.clinicalscientists.cn/freestatistics). A two-sided *p*-value of < 0.05 was considered statistically significant.

## Results

### Characteristic comparison between EIAS patients and controls

The study comprised 120 patients with diabetes mellitus (mean age 51.3 ± 17.5 years; 56.7% male). Based on diagnostic criteria, 37 patients (30.8%) were classified as having EIAS, while 83 (69.2%) served as controls. Characteristic comparison is detailed in Table 1. EIAS patients were significantly older (60.1 ± 17.2 years vs. 47.3 ± 16.2 years; *p* < 0.001) and had a longer median diabetes duration (12.0 [8.0, 20.0] years vs. 6.0 [1.0, 12.5] years; *p* = 0.003). No significant differences were observed between groups for gender distribution, BMI, type of diabetes, smoking status, or alcohol status.

**Table 1:** Clinical characteristics of EIAS patients and controls.

Variables	Total (*n* = 120)	Control (*n* = 83)	EIAS (*n* = 37)	*p*-value
Age, years	51.3 ± 17.5	47.3 ± 16.2	60.1 ± 17.2	<0.001
Sex, *n* (%)				0.114
Male	68 (56.7)	51 (61.4)	17 (45.9)	
Female	52 (43.3)	32 (38.6)	20 (54.1)	
BMI, kg/m^2^	22.8 ± 3.3	22.5 ± 3.2	23.5 ± 3.5	0.146
Type of DM, *n* (%)				0.180
T1DM	11 (9.2)	8 (9.6)	3 (8.1)	
T2DM	97 (80.8)	64 (77.1)	33 (89.2)	
LADA	12 (10.0)	11 (13.3)	1 (2.7)	
Duration, years	9.0 (1.0, 15.2)	6.0 (1.0, 12.5)	12.0 (8.0, 20.0)	0.003
Dose of insulin, IU	26.0 (9.5, 42.0)	20.0 (0.0, 37.0)	37.0 (26.0, 49.0)	<0.001
Smoke, *n* (%)	45 (37.5)	35 (42.2)	10 (27)	0.114
Alcohol, *n* (%)	21 (17.5)	18 (21.7)	3 (8.1)	0.071
Insulin analog, *n* (%)				
Aspart	57 (47.5)	34 (41)	23 (62.2)	0.032
Glargine	46 (38.3)	28 (33.7)	18 (48.6)	0.121
Lispro	8 (6.7)	6 (7.2)	2 (5.4)	1.000
Detemir	13 (10.8)	7 (8.4)	6 (16.2)	0.217
Degludec	40 (33.3)	31 (37.3)	9 (24.3)	0.162
Premixed human insulin	20 (16.7)	9 (10.8)	11 (29.7)	0.010
Hypoglycemic drugs, *n* (%)				
Metformin	61 (50.8)	44 (53)	17 (45.9)	0.475
Glycosidase inhibitors	31 (25.8)	21 (25.3)	10 (27)	0.842
SGLT2 inhibitor	16 (13.3)	10 (12)	6 (16.2)	0.567
DDP4 inhibitors	13 (10.8)	9 (10.8)	4 (10.8)	1.000
Sulfonylureas	5 (4.2)	3 (3.6)	2 (5.4)	0.643
TZD	5 (4.2)	5 (6)	0 (0)	0.322
GLP-1RA	8 (6.7)	5 (6)	3 (8.1)	0.701

Data are shown as mean ± standard deviation (SD) or median (IQR) for continuous variables and proportions (%) for categorical variables. SGLT2, sodium-glucose cotransporter 2; DDP4, dipeptidyl-peptidase 4; TZD, thiazolidinedione; GLP-1RA, glucagon-like peptide-1 receptor agonists.

Regarding insulin therapy, the daily insulin dose was substantially higher in the EIAS group (median 37.0 [26.0, 49.0] IU vs. 20.0 [0.0, 37.0] IU; *p* < 0.001). The use of aspart insulin was significantly more prevalent in the EIAS group (62.2% vs. 41.0%; *p* = 0.032). Similarly, the use of premixed human insulin was significantly higher among EIAS patients (29.7% vs. 10.8%; *p* = 0.010).

### Increasing of fasting insulin to C-peptide ratio in EIAS patients

Laboratory parameters including platelet count, differential leukocyte subsets (neutrophils, monocytes, and lymphocytes), hepatic transaminases (ALT and AST), albumin (ALB), serum uric acid (SUA), renal function markers (BUN and creatine), lipid profiles (TC, TG, HDL-C, and LDL-C), fasting and 2-h postprandial blood glucose, and 2-h postprandial C-peptide demonstrated no significant intergroup differences (all *p* > 0.05). However, the EIAS cohort exhibited significantly lower HbA1c (8.3 ± 1.6% vs. 9.9 ± 2.5%; *p* = 0.001) and admission fasting glucose (9.8 ± 3.5 mmol/L vs. 12.3 ± 5.9 mmol/L; *p* = 0.021) than control patients. Renal assessment revealed markedly reduced eGFR (88.2 ± 22.3 vs. 105.1 ± 24.6 mL/min/1.73m^2^; *p* < 0.001) and elevated ACR (median 51.8 [8.1–114.6] vs. 14.8 [6.6–53.9] mg/g; *p* = 0.045) in EIAS patients, as detailed in Table 2.

**Table 2:** Laboratory parameters between EIAS patients and controls.

Variables	Total (*n* = 120)	Control (*n* = 83)	EIAS (*n* = 37)	*p*-value
Platelet, 10^9/L	222.1 ± 62.6	221.9 ± 62.5	222.7 ± 63.8	0.947
Neutrophils, 10^9/L	4.0 ± 1.5	3.9 ± 1.6	4.3 ± 1.4	0.129
Monocytes, 10^9/L	0.4 ± 0.1	0.4 ± 0.1	0.4 ± 0.1	0.886
Lymphocytes, 10^9/L	1.7 ± 0.7	1.7 ± 0.7	1.7 ± 0.7	0.619
ALT, IU/L	19.0 (13.5, 28.0)	17.0 (13.0, 28.0)	20.5 (15.0, 27.2)	0.489
AST, IU/L	22.0 ± 12.2	22.0 ± 13.7	22.0 ± 8.2	0.999
Albumin, g/L	44.5 ± 3.8	44.6 ± 3.4	44.3 ± 4.5	0.755
SUA, umol/L	294.1 ± 99.2	288.9 ± 107.4	305.6 ± 77.9	0.416
Urea, mmol/L	5.5 ± 2.1	5.5 ± 2.1	5.6 ± 2.0	0.761
Creatinine, mmol/L	65.7 ± 27.1	62.9 ± 28.1	72.1 ± 23.8	0.09
eGFR, mL/min·1.73m^2^	100.0 ± 25.1	105.1 ± 24.6	88.2 ± 22.3	<0.001
TC, mmol/L	4.6 ± 1.5	4.8 ± 1.6	4.3 ± 1.3	0.145
TG, mmol/L	1.4 (1.0, 2.1)	1.4 (1.0, 2.3)	1.4 (0.9, 1.7)	0.358
HDL-C, mmol/L	1.3 ± 0.3	1.3 ± 0.3	1.3 ± 0.4	0.914
LDL-C, mmol/L	2.7 ± 1.0	2.8 ± 1.0	2.5 ± 1.0	0.187
Fasting BG, mmol/L	11.5 ± 5.4	12.3 ± 5.9	9.8 ± 3.5	0.021
Fasting insulin, uU/mL	4.4 (2.1, 14.5)	3.1 (1.3, 6.2)	14.2 (5.7, 37.3)	<0.001
Fasting C peptide, ng/mL	0.9 (0.6, 1.6)	0.9 (0.6, 1.5)	1.2 (0.7, 1.9)	0.071
2hPBG, mmol/L	14.9 ± 4.7	14.9 ± 4.3	15.0 ± 5.5	0.902
2 h Postprandial insulin, uU/mL	11.1 (4.5, 23.1)	8.4 (3.6, 15.3)	19.7 (11.2, 43.8)	<0.001
2 h Postprandial C peptide, ng/mL	1.8 (1.2, 3.1)	1.8 (1.0, 3.0)	2.0 (1.4, 3.5)	0.267
HbA1c, %	9.4 ± 2.4	9.9 ± 2.5	8.3 ± 1.6	0.001
ACR, g/ug	17.1 (7.1, 83.5)	14.8 (6.6, 53.9)	51.8 (8.1, 114.6)	0.045
Fasting insulin/C-peptide ratio	3.9 (2.7, 12.0)	3.4 (2.3, 4.9)	9.8 (5.5, 42.2)	<0.001

Data are shown as mean ± standard deviation (SD) or median (IQR) for continuous variables and proportions (%) for categorical variables. ALT, alanine aminotransferase; AST, aspartate aminotransferase; SUA, serum uric acid; eGFR, estimated glomerular filtration rate; TC, total cholesterol; TG, triglyceride; HDL-C, high-density lipoprotein cholesterol; LDL-C, low-density lipoprotein cholesterol; 2hPBG, 2-h postprandial blood glucose; HbA1c, glycated hemoglobin; ACR, albumin-to-creatinine ratio.

**Figure 1:**
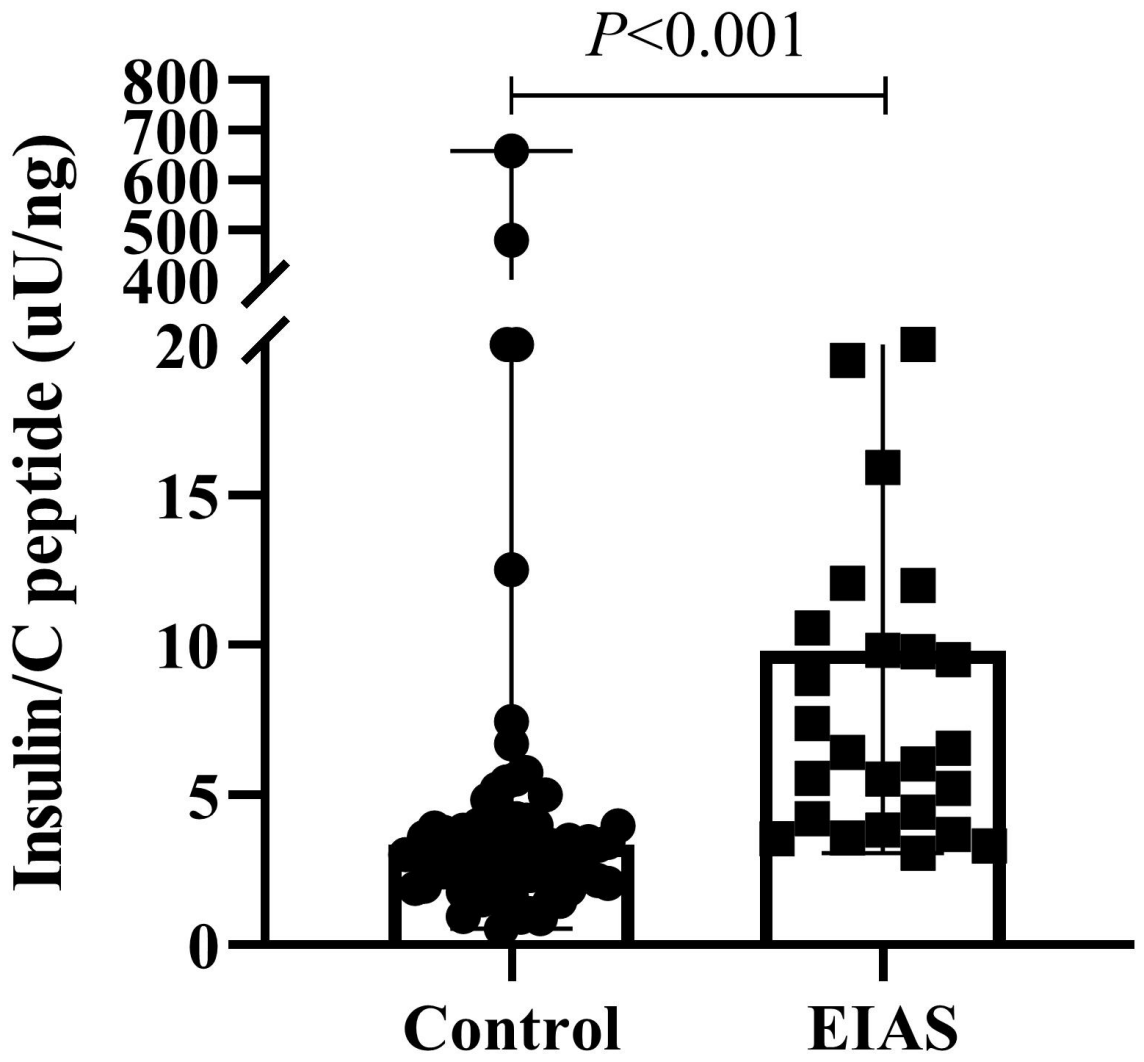
Fasting insulin/C-peptide ratio between IAA patients and controls.

Markedly elevated fasting insulin levels characterized the EIAS group (median 14.2 [5.7, 37.3] μU/mL vs. 3.1 [1.3, 6.2] μU/mL; *p* < 0.001), while fasting C-peptide showed a trend toward higher levels (median 1.2 [0.7, 1.9] ng/mL vs. 0.9 [0.6, 1.5] ng/mL; *p* = 0.071). Consequently, the fasting insulin/C-peptide ratio was significantly greater in EIAS patients (Figure 1). Postprandial insulin levels at 2 h were also substantially higher in the EIAS group (median 19.7 [11.2, 43.8] μU/mL vs. 8.4 [3.6, 15.3] μU/mL; *p* < 0.001, Table 2).

### The efficacy of fasting insulin/C peptide ratio in the diagnosis of EIAS

**Figure 2:**
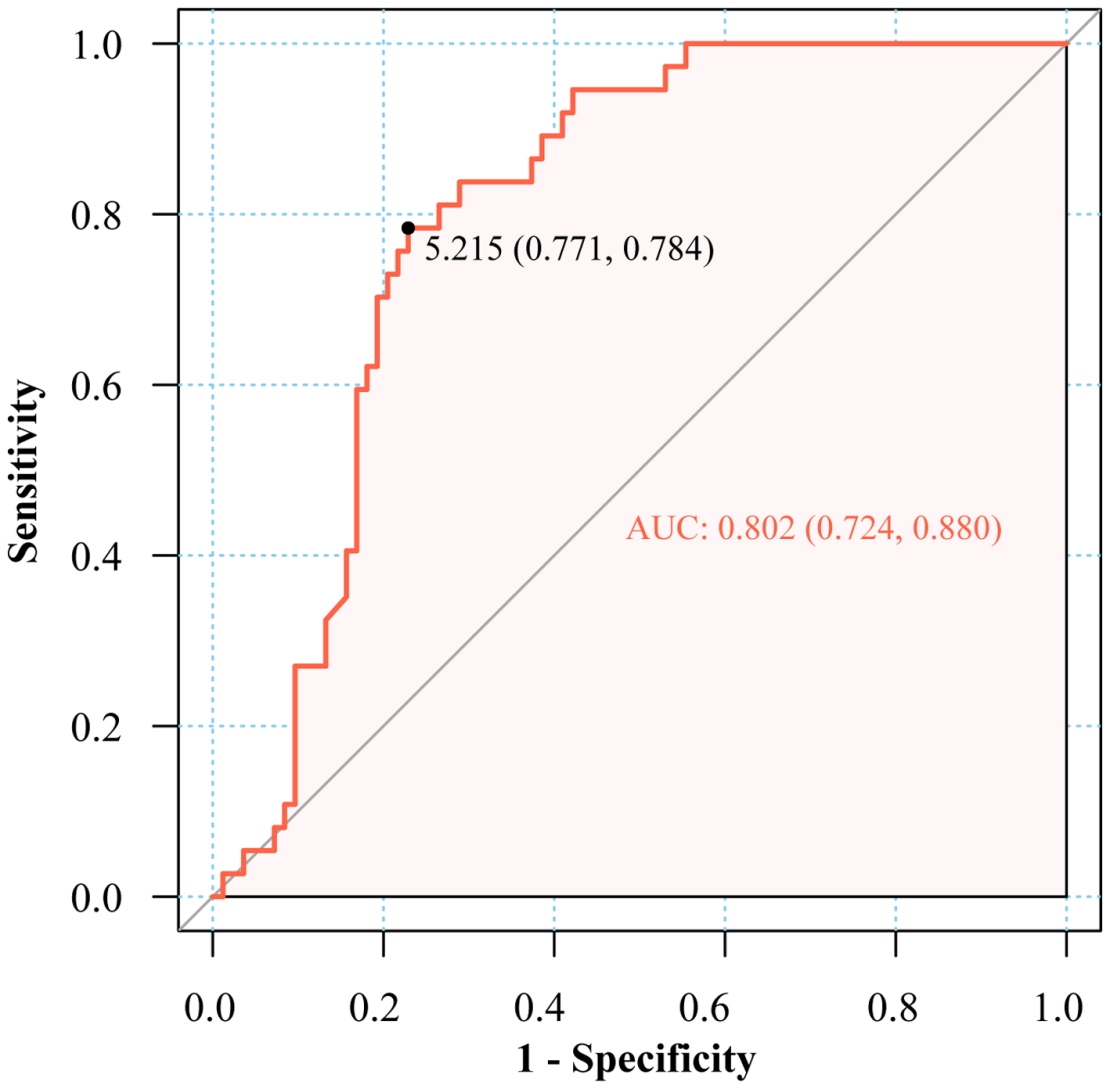
ROC of fasting insulin to C-peptide ratio in the diagnosis of EIAS.

ROC analysis identified the fasting insulin/C-peptide ratio as a favorable predictor in the diagnosis of EIAS, with an area under the curve (AUC) of 0.802 (95% CI: 0.724–0.880). The optimal diagnostic cutoff value, determined by maximizing the Youden index, was established at 5.215 μU/ng, corresponding to 78.4% sensitivity and 77.1% specificity (Figure 2).

## Discussion

To the best of our knowledge, this study first established that the fasting insulin-to-C-peptide ratio serves as a clinically viable biomarker for EIAS detection. Our analysis of 120 diabetic patients identified 37 EIAS cases (30.8%), revealing distinct clinical characteristics including advanced age, prolonged diabetes duration, and elevated daily insulin requirements compared to those non-IAS patients. No significant intergroup differences emerged regarding gender distribution, BMI, diabetes types, smoking, or alcohol status. Notably, aspart insulin and premixed human insulin formulations were utilized more frequently among EIAS patients, aligning with prior observations (4, 11). Supporting this finding, Nishimura et al. also identified protamine-containing insulin preparations and extended insulin therapy duration as significant risk factors for insulin autoantibody development (12).

The precise prevalence and incidence of IAS remain uncertain. The majority of reported cases originate from Asia, particularly Japan, where studies estimate a prevalence of 0.017 cases per 100,000 individuals (13). Retrospective data from Korea suggest a prevalence of 6% among a cohort of 84 patients undergoing investigation for endogenous hyperinsulinemic hypoglycemia at a referral center (14). However, epidemiological data from China are scarce. A systematic review of English and Chinese literature on IAS from 1970 to 2020 identified only 122 reported patients (4), highlighting the urgent need for nationwide epidemiological studies in China. To address this knowledge gap, we identified 37 cases of EIAS (30.8%) among 120 diabetic patients in our cohort. This prevalence is substantially higher than previously reported rates. This discrepancy may be primarily attributable to selection bias, as our study exclusively recruited patients with available IAA test results. Patients without clinical suspicion of IAS would typically not undergo this testing.

The pathogenesis of IAS centers on insulin–autoantibody complex formation, driving glucometabolic dysregulation through a biphasic mechanism. Initially, autoantibodies impede insulin binding during postprandial periods, potentially causing transient hyperglycemia. Subsequently, insulin is released from the complexes independently of glucose levels, inducing hypoglycemic episodes (2). Consistent with established pathophysiology, the EIAS cohort in this study exhibited markedly elevated fasting and postprandial insulin concentrations alongside significantly reduced HbA1c and fasting glucose levels, suggesting dysregulated insulin dissociation from insulin–autoantibody complexes rather than physiological secretion patterns. Fasting C-peptide showed a trend toward higher levels in the EIAS cohort, in contrast to the suppression typically induced by exogenous insulin administration. Although alterations in hyperglycemia and hypoglycemia could facilitate the identification of autoimmune hypoglycemia caused by EIAS, early-stage diagnostic challenges still persist. Due to the symptomatic overlap with insulinoma or excessive hypoglycemic agents, the current gold standards of IAS diagnosis require complex antibody testing and pancreatic imaging (15). Notwithstanding the costly and time-intensive nature of autoantibody assays, IAA assays have been reported to demonstrate variable diagnostic accuracy (73.6 to 79.0%) depending on assay methodology (16). Furthermore, IAA detection suffers from limited specificity due to cross-reactivity with exogenous insulin (2), while diagnostic delays inherent to these methods may increase patients’ hypoglycemia risk.

Hyperinsulinemic hypoglycemia remains the diagnostic cornerstone, while emerging evidence supports the insulin/C-peptide molar ratio as a viable screening alternative (4, 10). Nevertheless, standardized diagnostic thresholds remain undefined. Some studies have directly adopted an insulin/C-peptide molar ratio of > 1 as one of the diagnostic criteria for IAS (10). Pancreatic beta-cells secrete insulin and C-peptide in equimolar proportions. However, these molecules exhibit distinct half-lives: approximately 5–10 min for insulin and 30–35 min for C-peptide. Consequently, the physiological insulin-to-C-peptide molar ratio is typically less than 1 (2). The elevated fasting insulin/C-peptide ratio in EIAS patients can be attributed to the unique pathophysiology of insulin–autoantibody complexes. In EIAS, exogenous insulin binds to autoantibodies, forming complexes that significantly prolong insulin’s half-life, from minutes to several hours or days, while C-peptide clearance remains relatively unchanged. This dissociation in clearance kinetics results in disproportionately high circulating insulin relative to C-peptide, especially in the fasting state when endogenous secretion is minimal. Thus, the fasting insulin/C-peptide ratio serves as a functional biomarker that captures the underlying immune-mediated insulin dysregulation characteristic of EIAS, distinguishing it from other causes of hyperinsulinemic hypoglycemia (2, 17). However, other studies have reported inconsistent findings. For example, one study included 16 patients with IAA positive and showed that only 9 patients exhibited a ratio of >1, whereas 6 patients showed a ratio <1 (18). Consequently, the insulin-to-C-peptide molar ratio may also exceed 1 in exogenous insulin administration, as hypoglycemia suppresses endogenous insulin secretion, resulting in disproportionately low C-peptide levels (2). It has been proposed that an insulin-to-C-peptide molar ratio of >6 during hypoglycemia may serve as a useful indicator for defining autoimmune hypoglycemia caused by EIAS (10), though the limited sample size (*n* = 23) warrants further validation. In our cohort, this metric showed inconsistent elevation among confirmed EIAS cases, and the fasting insulin-to-C-peptide ratio (AUC 0.802) demonstrated enhanced clinical utility, with an established threshold of 5.215 μU/ng that accounts for diabetes-related insulin resistance, thereby enhancing its diagnostic applicability.

The precise mechanisms underlying EIAS remain incompletely elucidated. Current evidence implicates HLA-mediated immune dysregulation, particularly DRB1*04:06 allelic predominance, initiated by sulfhydryl-containing agents (methimazole, and alpha-lipoic acid, for example) (19, 20). Notably, emerging data characterize EIAS patients by a distinct autoimmune phenotype, evidenced by elevated frequencies of concomitant autoimmune disorders, food or drug hypersensitivities, and positive antinuclear antibodies (10), even though some cases can be a solitary manifestation of autoimmunity (2). This immunological profile suggests that individuals with pronounced autoimmune dysregulation may exhibit heightened susceptibility to autoimmune hypoglycemia following exogenous insulin exposure.

Fortunately, EIAS typically follows a self-limiting clinical course with a favorable prognosis due to substantial spontaneous remission rates (18, 20). Management primarily centers on hypoglycemia prevention through dietary modifications. Pharmacological intervention becomes necessary in severe conditions, using either insulin secretion suppressants, such as somatostatin analogs and diazoxide, or anti-immune therapy, such as glucocorticoids, azathioprine, and rituximab (2, 21). Discontinuing or substituting the causative insulin formulation typically resolves recurrent hypoglycemia concurrent with declining plasma insulin levels and IAA titers.

Several limitations of this study warrant consideration. First, the monocentric design may limit the generalizability of our findings to other populations or clinical settings. Second, the study cohort consisted exclusively of Han Chinese individuals, which restricts extrapolation to other ethnic groups. Third, the absence of non-diabetic controls and patients with classic IAS precludes comparative analyses across broader hypoglycemia etiologies. Fourth, the retrospective nature of the study introduces potential selection bias, particularly as IAA testing was performed based on clinical suspicion. Finally, the proposed cutoff for the fasting insulin/C-peptide ratio requires external validation in independent cohorts to confirm its diagnostic robustness.

In conclusion, the fasting insulin/C-peptide ratio offers a practical and economically feasible screening tool that could significantly streamline the diagnostic pathway for EIAS. Compared to specialized immunoassays and pancreatic imaging, this ratio leverages routinely available laboratory measures, reducing both cost and turnaround time. As highlighted in recent discussions on biomarker implementation in autoimmune diagnostics and cost-effective laboratory medicine strategies (22, 23), such accessible biomarkers can enhance early detection in resource-limited settings and reduce diagnostic delays.

## Data Availability

The raw data supporting the conclusions of this article will be made available by the authors, without undue reservation.
